# Functional Characterization of Zebrafish (*Danio rerio*) Bcl10

**DOI:** 10.1371/journal.pone.0122365

**Published:** 2015-04-07

**Authors:** Pellegrino Mazzone, Ivan Scudiero, Angela Ferravante, Marina Paolucci, Luca E. D’Andrea, Ettore Varricchio, Gianluca Telesio, Chiara De Maio, Maddalena Pizzulo, Tiziana Zotti, Carla Reale, Pasquale Vito, Romania Stilo

**Affiliations:** 1 Biogem, Via Camporeale, Ariano Irpino (AV), Italy; 2 Dipartimento di Scienze e Tecnologie, Università del Sannio, Via Port’ Arsa 10, Benevento, Italy; 3 Università di Napoli “Federico II”, Napoli, Italy; INRA, FRANCE

## Abstract

The complexes formed by BCL10, MALT1 and specific members of the family of CARMA proteins (CBM complex), have recently focused much attention because they represent a central hub regulating activation of the transcription factor NF-κB following various cellular stimulations. In this manuscript, we report the functional characterization of a Danio rerio 241 amino acids polypeptide ortholog of the Caspase recruiting domain (CARD)-containing protein BCL10. Biochemical studies show that zebrafish Bcl10 (zBcl10) dimerizes and binds to components of the CBM complex. Fluorescence microscopy observations demonstrate that zBcl10 forms cytoplasmic filaments similar to that formed by human BCL10 (hBCL10). Functionally, in human cells zBcl10 is more effective in activating NF-κB compared to hBCL10, possibly due to the lack of carboxy-terminal inhibitory serine residues present in the human protein. Also, depletion experiments carried out through expression of short hairpin RNAs targeting hBCL10 indicate that zBcl10 can functionally replace the human protein. Finally, we show that the zebrafish cell line PAC2 is suitable to carry out reporter assays for monitoring the activation state of NF- kB transcription factor. In conclusion, this work shows that zebrafish may excellently serve as a model organism to study complex and intricate signal transduction pathways, such as those that control NF-κB activation.

## Introduction

NF-κB is an inducible and ubiquitously expressed transcription factor for genes involved in immune and inflammatory responses, cell survival, cell adhesion, differentiation, and growth [1, 2]. Given that NF-κB transcribes genes that generally control both innate and acquired immune response and genes that play a positive effect on cell survival and proliferation, disregulation of the mechanisms controlling its activation often results in immunoproliferative and inflammatory phenotypes [1, 2]. A paradigmatic example of this is given by the human CARD-containing protein BCL10, a 233 amino acids protein initially identified by functional cloning approach from mucosa-associated lymphoid tissue (MALT) lymphoma cells [3, 4]. As a result of a translocation, in a subset of MALT B cell lymphomas BCL10 was overexpressed, resulting in an altered, constitutive activation of NF-κB that was eventually responsible for the neoplastic transformation [3, 4]. At the same time, BCL10 was independently cloned in other laboratories for its ability to activate the transcription factor NF-κB [5–10].

Genetic disruption of the BCL10 locus in murine strains results in immunodeficiency, having these genetically modified mice profound defects in humoral and cellular immune responses [11]. In fact, following antigen stimulation on B and T lymphocytes, BCL10 is indispensable for NF-κB activation, whose transcriptional activity is required for proper lymphocytes activation and proliferation [11].

The biological function of BCL10 is explicated through formation of the CBM complex, a molecular complex that includes one of three members of the family of CARMA proteins and the protein MALT1 [12]. The three CARMA proteins, CARMA1, 2 and 3, constitute in fact a family of proteins conserved across many species which are characterized by the presence of different functional domains shared by all members of the family [13–18]. Functionally, all three CARMA proteins are able to associate BCL10 through an homophilic interaction between the corresponding CARD domains, and cooperate with BCL10 to induce the transcriptional activity of NF-κB [13–18].

Recently, extensive analysis of the zebrafish (*Danio rerio*) genome have reported the presence of several CARD domain containing proteins encoded by the genome of this organism, including NOD1 and NOD2, RIPK2, PYCARD, CARD9, CARMA1, CARMA2, CARMA3 and BCL10 [19–22]. However, the functional analysis of these proteins is still incomplete. In this work, we report the functional characterization of the BCL10 ortholog in zebrafish.

## Materials and Methods

### Ethics

All the procedures involving animals were conducted as indicated in the Italian National Guidelines (D.L. No. 116 G.U., suppl. 40, 18.2.1992, circolare No. 8, G.U. July 1994) and in the appropriate European Directives (EEC Council Directive 86/609, 1.12.1987), adhering to the Guide for the Care and Use of Laboratory Animals (United States National Research Council, 1996). All the in vivo experimental activities were approved by the Animal Ethics Committee (CESA) of Biogem (Italy).

### RNA extraction and cloning of zBcl10 full-length cDNA

Total RNA samples were extracted from whole 6-day larvae using Trizol RNA isolation reagent (GIBCO-BRL) according to the manufacturer’s instructions. 1 μg of total RNA was primed with oligo(dT) and reverse-transcribed using the QuantiTect Reverse Transcription Kit (Qiagen) according to the manufacturer’s instructions to generate a first-strand cDNA. Primers used to amplify zBcl10 were the following: forward 5’-ATGGATGTTACTCACCTG-3’ and reverse 5’-GACGTTTACGGAGACAAA-3’. PCR conditions were as follows: 98°C for 30 s, 30 cycles (98°C/5 s; 63°C/22 s; 72°C/30 s), and then 72°C for 5 min. Thermo Scientific® Phusion High-Fidelity DNA Polymerase (New England BioLabs) was used as amplifying polymerase, according to the manufacturer’s instructions. The RT-PCR product of the expected size was gel purified and cloned into pcDNA3 expression vector (Addgene), provided with an amino-terminal HA or FLAG epitope using standard cloning methodologies and confirmed by sequencing. Three independent clones deriving from three different PCR amplifications gave the same nucleotides sequence, corresponding to that deposited in GenBank with access number XM_002660692.

The expression vectors used in this study have been previously described [5, 23–27]

### Sequence analysis and phylogenic analysis of zBcl10

The zBcl10 protein sequence was analyzed by using the BLAST algorithm at the NCBI web site (http://www.ncbi.nlm.nih.gov/blast), and the multiple sequence alignment was created with ClustalW program (http://www.ebi.ac.uk/clustalw/) with default settings. Phylogenetic analyses with bootstrapping (100 replicates) were obtained by the Neighbor-joining method using complete deletion and the p-distance amino acid model in MEGA [28].

### Cell culture and transfections

HEK293 cells were maintained in Dulbecco's modified Eagle's medium (DMEM) supplemented with 10% FBS. The PAC2 fibroblast line, isolated from 24-h post-fertilization zebrafish embryos, was kindly provided by Dr. Isidoro [29]. PAC2 cells were cultured under standard conditions (28°C; room atmosphere) in Leibovit's L-15 medium (Sigma-Aldrich) supplemented with 40% of heat-inactivated fetal bovine serum. HEK293 cells were transfected by standard calcium/phosphate procedure using 10 μg of plasmidic DNA. PAC2 cells were transfected with Lipofectamine 2000 (Invitrogen) following the manufacturer's protocol.

Short hairpin RNAs targeting hBCL10 were the following:

shBCL10 #2: 5’-CCACCAGATCTACAGTTAGAACTCGAGTTCTAACTGTAGATCTGGTGGC-3’

shBCL10 #3: 5’-CCTTAAGATCACGTACTGTTTCTCGAGAAACAGTACGTGATCTTAAGG-3’

shBCL10 #4: 5’-CTTGTCGAACATCAAGTAGAACTCGAGTTCTACTTGATGTTCGACAAG-3’

shBCL10 #5: 5’-GTTGAATCTATTCGGCGAGAACTCGAGTTCTCGCCGAATAGATTCAAC-3’

Retroviral infections were carried out as previously described [30, 31].

### Luciferase and β-galactosidase assays

To assess for NF-κB activation, HEK293 were co-transfected in 6-well plates with 0.2 μg of pNF-κB-luc (Clontech), 0.1 μg of pRSV-βGal (Addgene) plus each expression plasmid. When necessary, the total amount of transfected plasmidic DNA (2 μg) was kept constant by adding empty vector. pNF-κB-luc encodes the firefly luciferase reporter gene under the control of a minimal (m)CMV promoter and tandem repeats of the NFκB transcriptional response element. The plasmid RSV-βGal, expressing β-galactosidase, was added to the transfection mixture in order to normalize for the efficiency of transfection. After transfection and treatments, luciferase activity was determined with Luciferase Assay System (Promega). For measurement of β-galactosidase activity, 20 μl of cell lysates diluted 100-fold with 0.1 M potassium phosphate buffer was mixed with 200 μl of Galactone (Tropix, Bedford, MA, USA) that was diluted 100-fold with 0.1 M potassium phosphate and 1 mM magnesium chloride, pH 7.8, for 1 hr at room temperature. Then, β-galactosidase activity was measured after addition of 300 μl of Emerald (Tropix). Luciferase activity was normalized on β-galactosidase activity and expressed in arbitrary units.

### Immunoblot analysis and coprecipitation

Cell lysates were made in lysis buffer (150 mM NaCl, 20 mM Hepes, pH 7.4, 1% Triton X-100, 10% glycerol) and a mixture of proteases inhibitors (Protease Inhibitor Cocktail, Roche) according to the manufacturer’s instructions. Proteins were separated by SDS–PAGE, transferred onto nitrocellulose membrane, and incubated with primary antibodies followed by horseradish peroxidase-conjugated secondary antibodies (Amersham Biosciences). Blots were developed using the ECL system (Amersham Biosciences). For co-immunoprecipitation experiments, cells were lysed in lysis buffer and immunocomplexes were bound to protein A/G (Amersham Biosciences) for 2 hrs at 4°C. immunocomplexes were extensively washed, resolved by SDS–PAGE, and analyzed by immunoblot assay. Sources of antisera and monoclonal antibodies were the following: anti-FLAG, anti-β-Actin, Sigma; anti-HA and anti-BCL10 (H-197 *SC5611*, generated against an epitope corresponding to amino acids 1–197 of human BCL10), Santa Cruz Biotechnology. The calf-intestinal alkaline phosphatase was purchased from Roche.

### Zebrafish tissues immunoblot analysis

6–9 months male zebrafish were euthanized and dissected. Proteic extracts from selected organs were prepared using Nonidet P-40 lysis buffer (1% v/v) Nonidet P-40, 150 mm NaCl, 50 mm Hepes, pH 7.4, 5 mm EDTA, 10% (v/v) glycerol, and complete protease inhibitor mixture (Roche). After homogenization and centrifugation (13,000 × g, 15 min, 4°C), protein concentration of supernatant was determined by BCA protein assay (Pierce). A 15 μg sample of whole cell extract was separated on SDS–polyacrylamide gel and transferred to membranes. Filters were blocked for 2 hrs in 3% nonfat dry milk in phosphate-buffered saline (PBS) with 0.3% Tween 20. Western blot analysis was performed using a rabbit anti-BCL10 antisera, followed by horseradish-peroxidase-conjugated mouse anti-rabbit antibody (Amersham Biosciences). Signal was developed using an enhanced chemiluminescence method (Amersham Biosciences) according to the manufacturer’s instructions.

### Immunofluorescence

1 x 10^4^ HEK293 were grown to 50% confluence and transfected in six-well chamber slides (Falcon). Sixteen hours after transfection, cells were fixed in 4% paraformaldehyde for 15 min at room temperature and then permeabilized in PBS/0.1% Triton X-100. Cells were incubated for 30 min in 5% FCS–PBS with anti-FLAG antibody (Sigma-Aldrich) followed by several washes with 5% FCS–PBS, and then incubating for 30 min with secondary antibody in 5% FCS–PBS. All steps were done at room temperature.

## Results

### zBcl10 characterization

**Fig 1:**
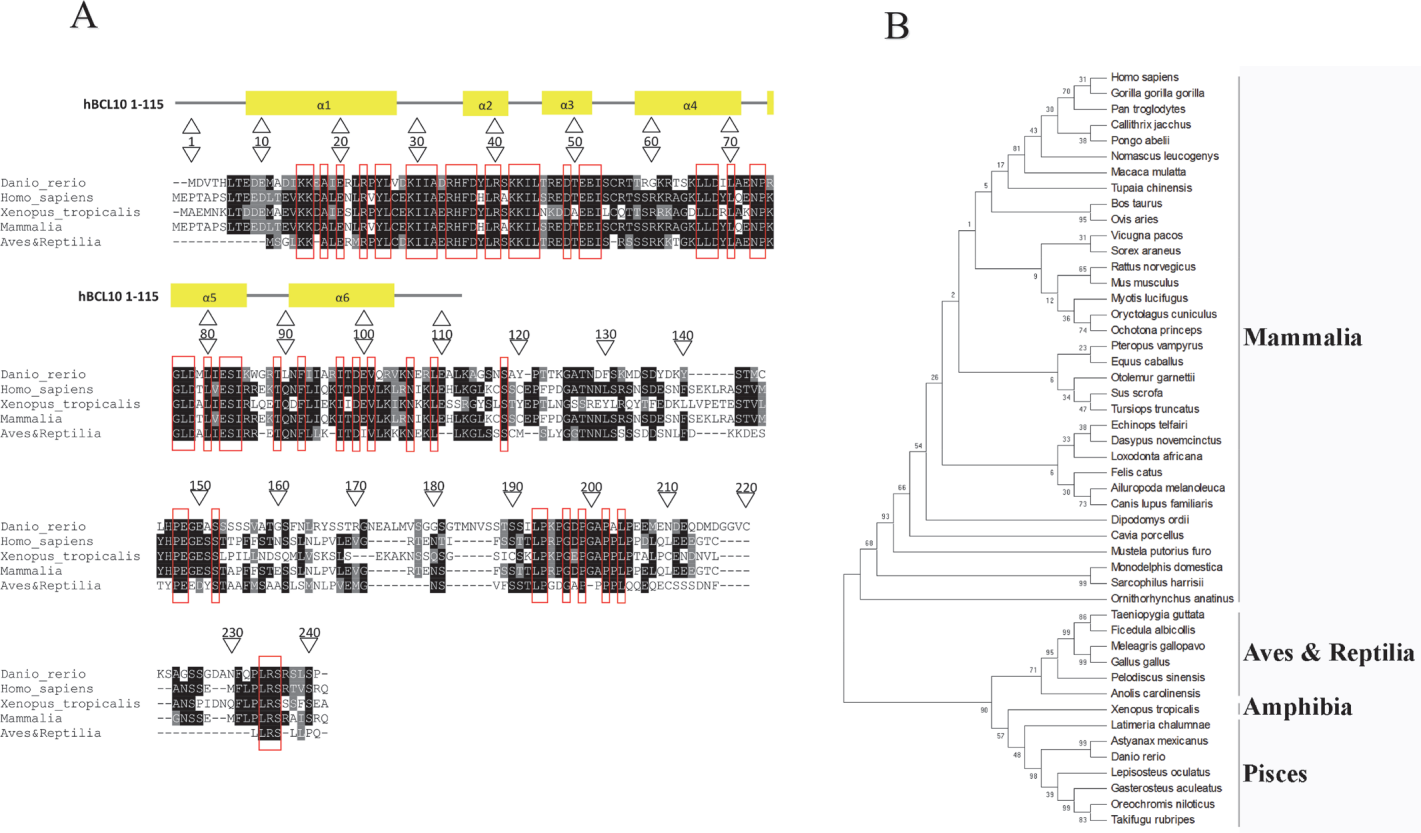
Alignment and phylogenetic tree of zBcl10. A) Alignment of zBcl10 and human BCL10 with the consensus sequence generated by aligning the BCL10 sequences of fish, birds and reptiles, and mammals. The Xenopus tropicalis sequence is the only amphibian BCL10 sequence available. The alignment was done using ClustalW. Printout from multiple-aligned sequences and consensus sequences calculation were done with BOXSHADE. The black background designates identical amino acids, the gray background conservative substitutions. The red rectangles indicate amino acids conserved in all sequences examined. At the top of the alignment, the six alpha helix regions of the CARD are shown. The sequences used for alignment and generation of the consensus sequence are available in Supplementary Material. B) Phylogenetic tree analysis of BCL10 proteins. Phylogenetic analyses with bootstrapping (100 replicates) were obtained by the Neighbor-joining method using complete deletion and the p-distance amino acid model in MEGA [28]. The percentage of replicate trees in which the associated taxa clustered together in the bootstrap test (100 replicates) are shown next to the branches. The sequences used for phylogenetic tree generation are available in Supporting Information (S1 Table).

The determination and the analysis of the zebrafish genome revealed the existence, in zebrafish, of a sequence similar to that encoding for the human protein BCL10 [19–22]. Thus, a full length cDNA corresponding to the GenBank sequence XM_002660692, putatively encoding for a zebrafish Bcl10 protein, was successfully amplified by RT-PCR from total zebrafish mRNA. It encodes for a protein of 241 amino acids with a predicted molecular mass of 26 kDa (Table 1 and Fig. 1A). The overall amino acidic identity of zBcl10 to human BCL10 (hBCL10) is 46% (Table 1). The major amino acidic differences between the two proteins are located at the carboxy-terminal of the polypeptides, whereas the amino-terminal CARD domains of zBcl10 (amino acids 6–113) and hBCL10 (amino acids 8–115) share 62% identity (Table 1). Sequence analysis shows that several residues that have been demonstrated to be necessary for the biological activity of hBCL10, namely I33, R36, D39, L41, R42, E50, E53, E54, R62 and G78 [4–6, 23, 32, 33], are conserved in zBcl10. On the other hand, residues S136, S141 and S144, which are implicated in attenuation of hBCL10 signaling [34], are not conserved in zBcl10 (Fig. 1A). A phylogenetic tree was constructed by the neighbor-joining method using MEGA [28], and it shows that zBcl10 clusterizes within the fish BCL10 sequences (Fig. 1B).

**Table 1:** Amino acidic similarity between hBCL10 and zBcl10 in the entire protein and in the CARD domains (hBCL10 8–115 and zBcl10 6–113).

**Species**	**Protein**	**Length**	**Identities**	**Positives**	**Gaps**
*Danio rerio*	zBcl10	241	112/241 (46%)	178/241 (74%)	28/241 (12%)
*Homo sapiens*	hBCL10	233			
*Danio rerio*	zBcl10 6–113	108	67/108 (62%)	90/108 (83%)	0/108 (0%)
*Homo sapiens*	hBCL10 8–115	108			

**Fig 2:**
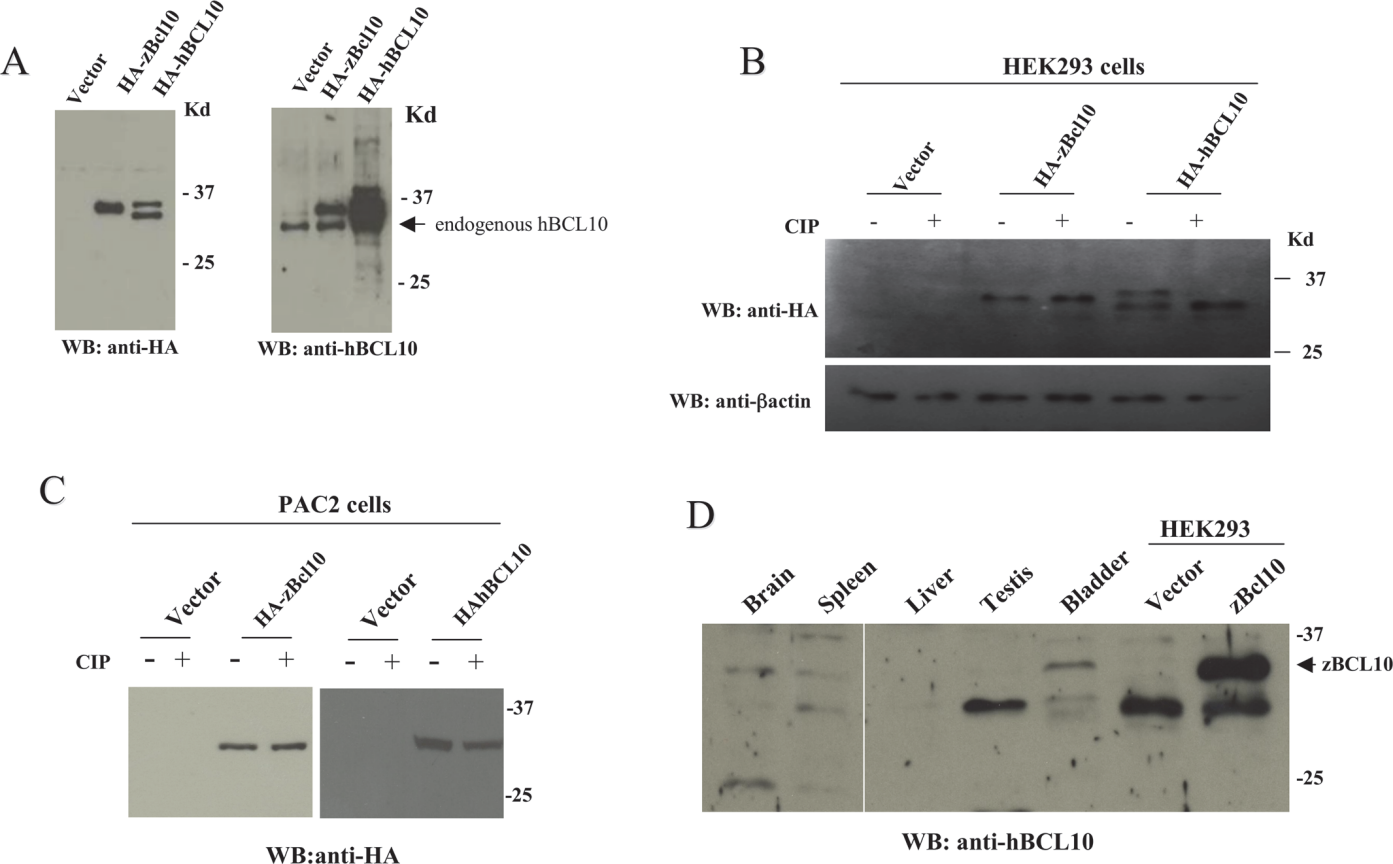
zBcl10 is not phosphorylated. A) Immunoblot analysis of lysates from HEK293 cells transfected with and expression vector empty (*vector*) or expressing HA-zBcl10 and HA-hBCL10. 24 hrs after transfection, cell lysates were prepared, separated by SDS/PAGE, and analyzed by immunoblot assay probed with anti-HA (left panel) and anti-BCL10 (right panel) antisera. An arrow indicates hBCL10 endogenously expressed by HEK293 cells. B) Cell lysates from HEK293 transfected cells were prepared as in A). Were indicated, before SDS/PAGE separation cell lysates were treated with 10 units of calf intestinal phosphatase (CIP) for 30 min at 37°C. C) The same experiment shown in B) was carried out in the embryonic zebrafish fibroblast cell line PAC2. D) Immunoblot analysis of proteic extracts from zebrafish organs probed with an anti-hBCL10 antisera. Cell extract from HEK293 cells transfected with zBcl10 was used as a positive control (arrow).

When analyzed in immunoblot assay, HA-tagged zBcl10 expressed in mammalian cells migrates as a 35 kDa protein (Fig. 2A, left panel). Interestingly, an anti-BCL10 antisera generated against the human protein (see Material and Methods), also recognizes the zebrafish counterpart (Fig. 2A, right panel). In these immunoblot experiments, we noticed that while hBCL10 migrates as a doublet on SDS-PAGE due to phosphorylation of the protein [15, 35], zBcl10 occurs as a single band, suggesting that zBcl10 is not target of phosphorylation events. In fact, experiments conducted by making use of a phosphatase confirmed this possibility (Fig. 2B). Lack of zBcl10 phosphorylation is likewise observed also when this protein is expressed in the embryonic zebrafish fibroblast cell line PAC2 (Fig. 2C). When transfected in these cells, hBCL10 migrates as a single band on SDS-PAGE, suggesting that phosphorylation of BCL10 does not occur in zebrafish (Fig. 2C). Finally, immunoblot experiments carried out on proteic lysates extracted from different zebrafish organs indicated that zBcl10 is endogenously expressed in bladder, spleen and brain, but not in testis and liver (Fig. 2D). In several tissues, including spleen and testis, the anti-hBCL10 antisera used in this study also recognizes a band of lower molecular weight, which could either be the result of a cross-reaction, or represent a shorter form of zBcl10, possibly arising from alternative splicings of zBcl10 mRNAs and/or post-translational modifications of the protein.

**Fig 3:**
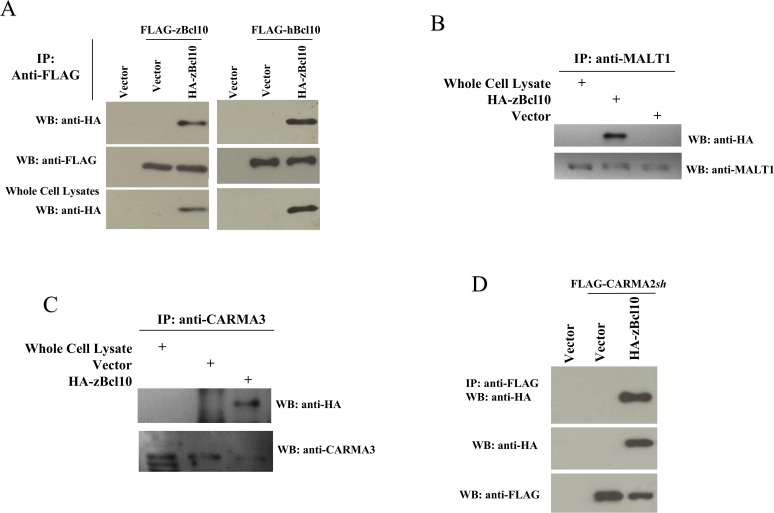
zBcl10 dimerizes and binds to CBM proteins. A) HEK293 cells were transiently cotransfected with FLAG-tagged and HA-tagged version of zBcl10 and hBCL10 or empty vector (*vector*). 24 hrs later, cell lysates were immunoprecipitated with anti-FLAG mAb. Immunocomplexes were separated by SDS-PAGE and transferred onto membranes subsequently probed with anti-HA antisera. *B-C)* Lysates from HEK293 cells transfected with HA-zBcl10 were immunoprecipitated with anti-MALT1 (B) or anti-CARMA3 (C) and analyzed for coprecipitating HA-zBcl10. D) HEK293 cells were transiently cotransfected with HA-zBcl10 and FLAG-tagged CARMA2*sh* or empty vector. Lysates were immunoprecipitated with anti-FLAG mAb and analyzed for coprecipitating HA-zBcl10 by immunoblot assay.

In mammals, BCL10 has an essential role in the signal transduction pathway that leads to activation of the transcription factor NF-κB [11, 12]. hBCL10-mediated activation of NF-κB requires oligomerization of hBCL10, assembly of the CBM complex and the triggering of unconventional ubiquitination events [12, 36], which eventually result in the recruitment of the IKK complex [24]. Indeed, transfection experiments shown in Fig. 3 indicate that zBcl10 is able to oligomerize both with itself and with hBCL10 (Fig. 3A) when expressed in the human cell line HEK293. Furthermore, zBcl10 associates with human MALT1 (Fig. 3B), and with human CARMA3 and CARMA2*sh* isoform (Fig. 3C-D) [13, 18].

### zBcl10 activates NF-κB

**Fig 4:**
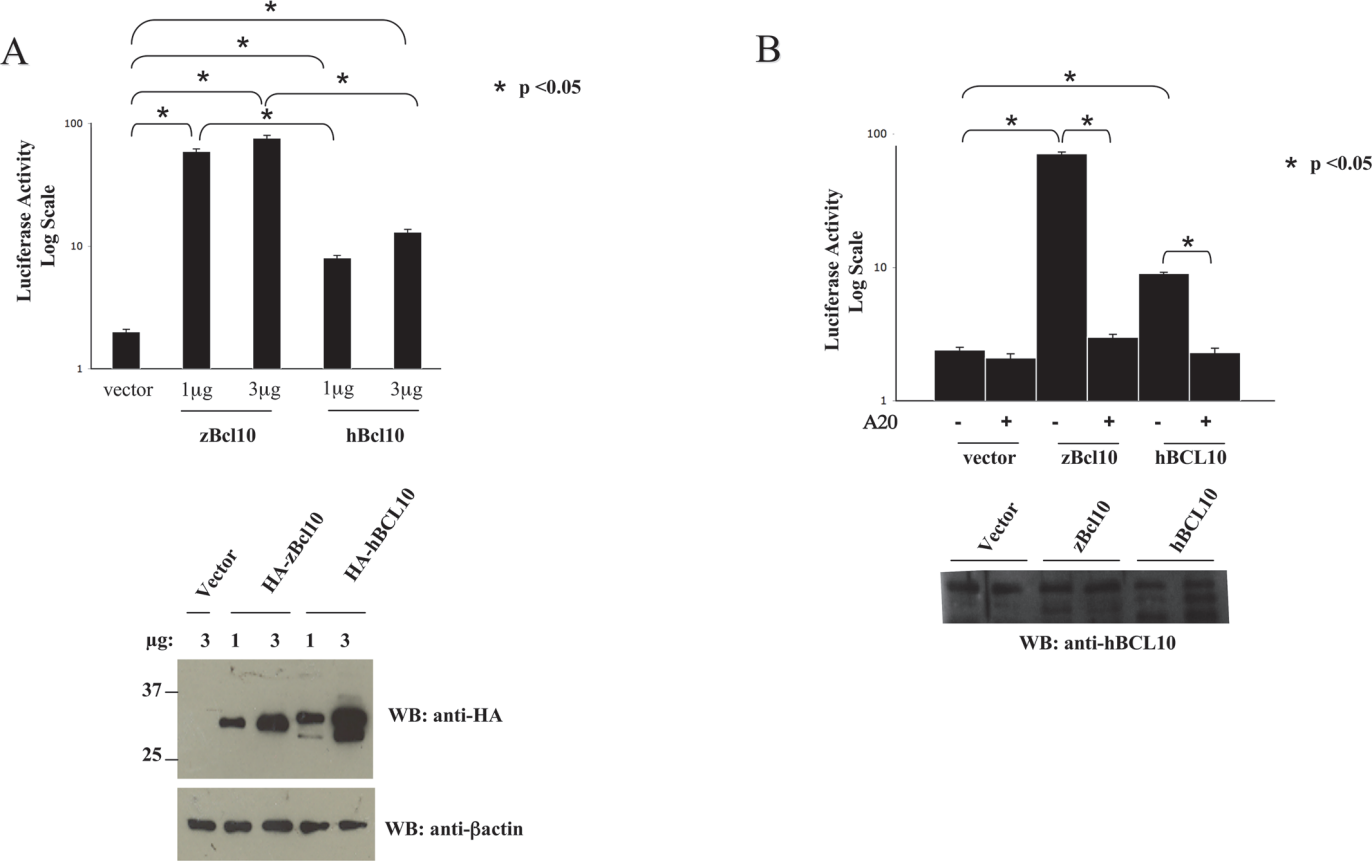
zBcl10 activates NF-κB. A) HEK293 cells were transiently cotransfected with an expression vector empy (*vector*) or encoding for the indicated polypeptides, together with pNF-κB-luc and pRSV-βgal reporter vectors. The total amount of transfected plasmidic DNA was maintained constant by adding empty vector. 16 hrs after transfection, cell lysates were prepared and luciferase activity was measured. In the bottom panels, a fraction of the cell lysates were analyzed by immunoblot to monitor protein expression. Data shown (mean + SEM, n = 9) represent relative luciferase activity normalized on β-galactosidase activity and is representative of six independent experiments done in triplicate. Statistical analysis was performed by Student's t test; a p value of <0.05 was considered significant, and is indicated with the symbol *. B) HEK293 cells were transiently cotransfected with an expression vector empy (*vector*) or encoding zebrafish and human BCL10 with or without the de-ubiquitinase A20. 16 hrs after transfection, cell lysates were prepared and luciferase activity was determined as in *A)*.

Next, we tested whether zBcl10 is able to activate NF-κB in mammalian cells using an NF-κB luciferase-based reporter plasmid, in which the firefly luciferase reporter gene is placed under the control of a minimal (m)CMV promoter and tandem repeats of the NFκB transcriptional response element (see Material and Methods section). The results of these experiments, shown in Fig. 4A, indicate that zBcl10 is significantly more effective than hBCL10 in activating NF-κB in mammalian cells. In fact, while expression of hBCL10 produces a luciferase activity about 8-10-fold higher compared to the empty vector, the luciferase activity produced by zBcl10 expression was at least 10-fold higher than that produced by hBCL10. As for hBCL10 [25, 37], zBcl10-induced NF-κB activation requires ubiquitination(s) events, since NF-κB activation is completely abrogated following co-expression of A20 de-ubiquitinase (Fig. 4B).

**Fig 5:**
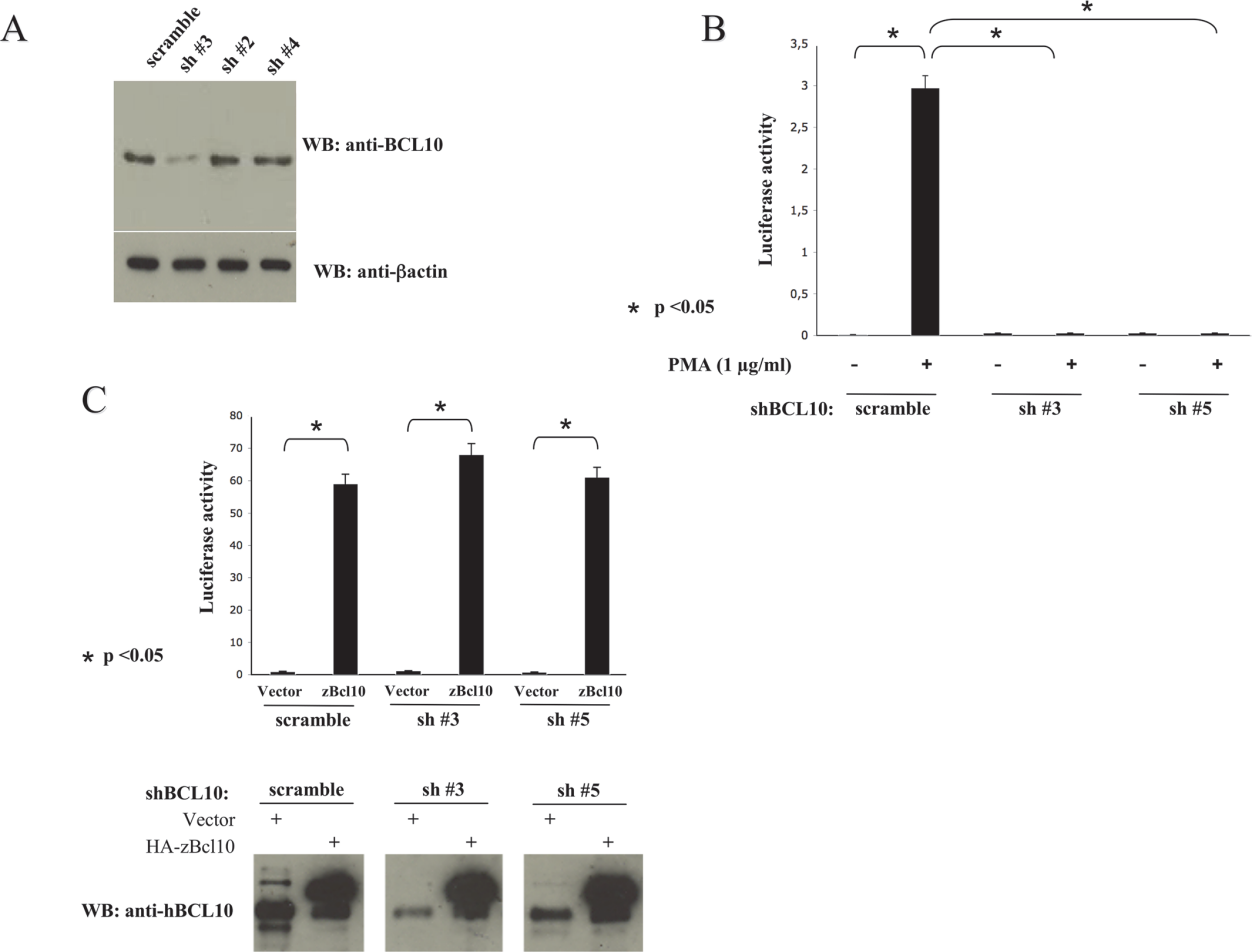
zBcl10 replaces hBCL10. A) HEK293 cells were infected with recombinant retrovirus expressing shRNAs targeting hBCL10. 72 hrs later, hBCL10 expression was monitored by immunoblot assay. B) NF-κB-driven luciferase activity in HEK293 cells silenced for hBCL10 and stimulated with PMA. C) NF-κB-driven luciferase activity in HEK293 cells silenced for hBCL10 and transfected with zBcl10. *Lower panel*: A fraction of the cell lysates were analyzed by an immunoblot assay probed with anti hBCL10 to monitor protein expression. Data shown (mean + SEM, n = 9) represent relative luciferase activity normalized on β-galactosidase activity and is representative of six independent experiments done in triplicate. Statistical analysis was performed by Student's t test; a p value of <0.05 was considered significant, and is indicated with the symbol *.

To exclude the possibility that NF-κB activation mediated by zBcl10 was due to its interaction and subsequent oligomerization of hBCL10, we abolished expression of hBCL10 in the human cell line HEK293 through retrovirus-mediated trasduction of short hairpin RNAs (shRNA) targeting hBCL10. As shown in Fig. 5A, introduction of hBCL10sh#3 in HEK293 cells results in a great reduction of BCL10 expression, whereas hBCL10sh#2 and hBCL10sh#4 had no significant effect on hBCL10 protein expression. A remarkable reduction of BCL10 expression was also observed when hBCL10sh#5 was used (data not shown). Depletion of hBCL10 in these cells abrogates their ability to activate NF-κB following exposure to phorbol-12-myristate-13-acetate (PMA) (Fig. 5B). However, introduction of zBcl10 in these hBCL10-depleted cells promptly activates NF-κB (Fig. 5C). Thus, zBcl10 can functionally replace hBCL10.

### zBcl10 forms filaments

**Fig 6:**
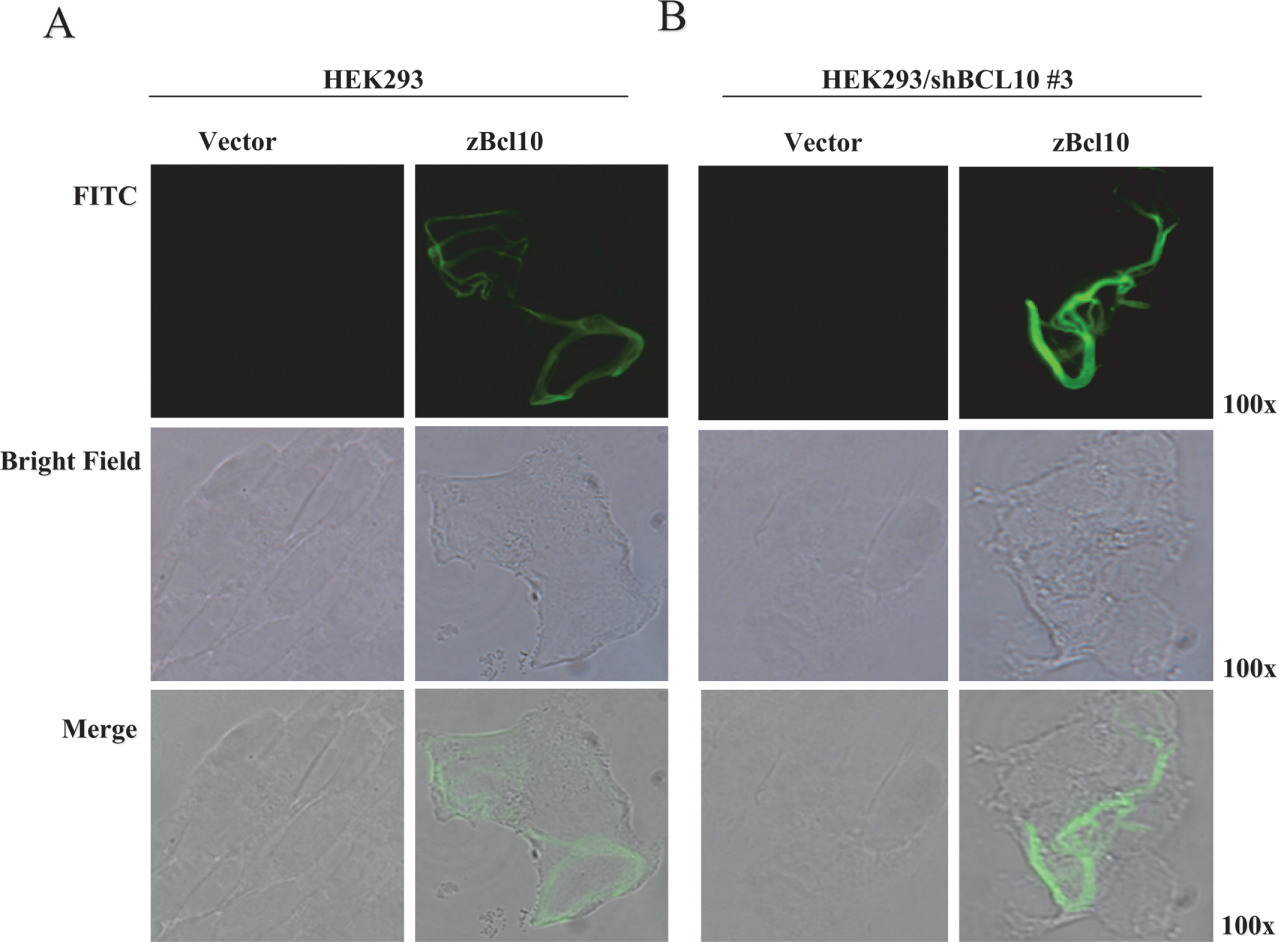
Subcellular localization of zBcl10. A) HEK293 cells were transfected with mammalian FLAG-tagged vector, empty (vector) or expressing zBcl10. 16 hrs after transfection, cells were stained with anti-FLAG mAb, followed by FITC-conjugated anti-mouse IgG. B) The same experiment described in A) was carried out in HEK293 cells infected with recombinant retrovirus expressing a shRNA targeting hBCL10. All photographs were taken at a 100X magnification.

Fluorescence microscopy experiments and structural studies have shown that the NF-κB activity produced by hBCL10 is regulated through formation of cytosolic filamentous structures [23, 33]. We therefore verified whether also zBcl10 is able to form such structures. As shown in Fig. 6A, assembly of filamentous structures is readily visible following expression of zBcl10 in mammalian cells. To exclude the possibility that the observed filamentous structures are not just precipitates of the over-expressed zBcl10 within the heterologous protein expressed in human cells, a similar experiment was conducted in hBCL10-depleted cells. As shown in Fig. 6B, zBcl10 forms filamentous structures even in the absence of hBCL10, thus confirming that the ability to form filaments is an intrinsic property of zBcl10.

**Fig 7:**
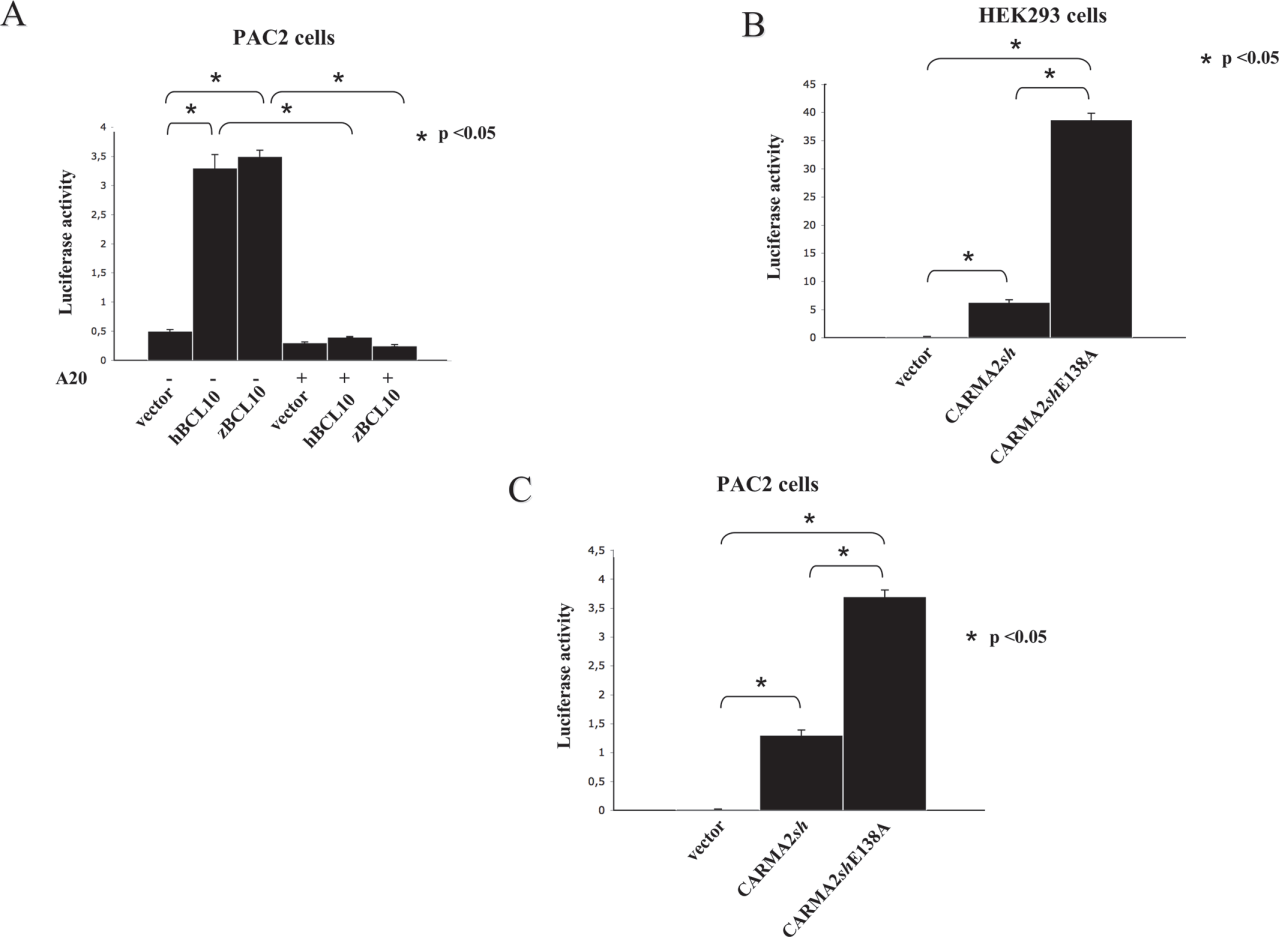
zBcl10 activates NF-κB in zebrafish cells. A) PAC2 cells were transiently cotransfected with expression vectors encoding for the indicated polypeptides, together with pNF-κB-luc and pRSV-βgal reporter vectors. The total amount of transfected plasmidic DNA was maintained constant by adding empty vector. 24 hrs after transfection, cell lysates were prepared and luciferase activity was measured. Data shown (mean + SEM, n = 9) represent relative luciferase activity normalized on β-galactosidase activity and is representative of six independent experiments done in triplicate. Statistical analysis was performed by Student's t test; a p value of <0.05 was considered significant, and is indicated with the symbol *. B, C) HEK293 and PAC2 cells were transiently cotransfected with expression vectors encoding for wt CARMA2*sh* or the psoriasis-linked mutants CARMA2*sh*E138A, together with pNF-κB-luc and pRSV-βgal reporter vectors and luciferase activity was determined as described in A).

Finally, we examined whether zBcl10 is able to activate NF-κB in zebrafish cells. For this, we used the the same NF-κB-luciferase assay we have used for mammalian cells (Figs.4–5). Indeed, although the NF-κB-luciferase reporter construct used in these experiments is optimized for mammalian cells, expressed in PAC2 cells both zBcl10 and hBCL10 significantly stimulate NF-κB-dependent luciferase activity, which is inhibited by A20 (Fig. 7A). This result gave us the possibility to verify if the zebrafish model can be used to study human pathological conditions resulting from altered CBM-mediated NF-κB activation. Thus, we expressed in both mammalian and PAC2 cells a mutant form of CARMA2*sh*, CARMA2*sh*E138A, which in humans is responsible for genetic psoriasis [38]. As previously shown [38], and confirmed in Fig. 7B, in the NF-κB-luciferase reporter assay CARMA2*sh*E138A induces hyperactivation of NF-κB compared to wt CARMA2*sh*. Since NF-κB transcribes genes that mediate the inflammatory response, such hyperactivation is thought to contribute to the manifestation of the psoriatic phenotype. Interestingly, hyperactivation of NF-κB following expression of CARMA2*sh*E138A was also observed in PAC2 cells, confirming the existence of common regulatory mechanisms for NF-κB activation.

## Discussion

There are several reasons that make particularly interesting the work here presented. The first one is represented by the possibility of using zebrafish as a model system to study the signal transduction pathways that modulate the activation state of the transcription factor NF-κB. Given the importance of this transcription factor in both normal cell biology and autoimmune, immunoproliferative and tumoral disorders, the possibility of using a model organism so flexible and informative such as zebrafish, certainly represents a field to massively explore further. This possibility is further supported by the extensive genomic knowledge on zebrafish we are acquiring in these days. Recently, one of these studies has revealed the presence of genes encoding for several putative CARD-containing proteins in the zebrafish genome, including the three CARMA proteins [20]. It would be certainly interesting to see whether ancillary proteins that have been demonstrated to modulate the activity of the CBM complex in mammals, such as USP9X [39], CKIP-1 [40], Net1 [41], p62 [42, 43], and various protein kinases and phosphatases [44–47], maintain a similar function in zebrafish.

Secondly, it is interesting to note that the serine residues with inhibitory function present in hBCL10 (S136, S141 and S144) [34] are not present in zBcl10. Thus, the negative regulation of BCL10 based on the serine phosphorylation seems to be a more recent evolutionary acquisition. This possibility is consistent with the greater ability of zBcl10 to activate NF-κB compared to hBCL10 (Fig. 4), and is further supported by the evidence that zBcl10, unlike hBCL10, does not appear to be target of phosphorylation reactions (Fig. 2).

Finally, the data presented here demonstrate a perfect conservation of the mechanisms through which BCL10 regulates the activation of NF-κB state, and thus concretely open the possibility of using zebrafish as a model system. Regarding this, it is certainly intriguing to note that the ubiquitination mechanisms required by hBCL10 to activate NF-κB are as well conserved in zBcl10 (Fig. 4). But, much more important, is the evidence here shows that mutant forms of genes that in humans cause diseases through the hyperactivation of NF-κB, such as the psoriasis-linked mutant CARMA2*sh*E138A, behave exactly the same way when analyzed in zebrafish (Fig. 7).

In summary, the present study has clearly demonstrated that the NF-κB signalling pathways regulated by BCL10 are conserved among vertebrates. Data presented will support further investigation of this intricate and fascinating pathway in zebrafish, which could represent a valuable *in vivo* model for the development of molecular tools capable of modulating the biological activity of BCL10 [48].

## Supporting Information

S1 Table List of sequences used for generation of the phylogenetic shown in Fig. 1B. (PDF)
