# Supplementary material for: Functional Characterization of Zebrafish (Danio rerio) Bcl10
Source: PLoS One. 2015 Apr 7;10(4):e0122365. doi: 10.1371/journal.pone.0122365 (PMC4388727; doi:10.1371/journal.pone.0122365)
Supplement: S1 Table — (PDF) [file pone.0122365.s001.pdf]

| Species | Identifier | |
| --- | --- | --- |
| | **Ensemble** | **GenBank** |
| Homo sapiens | ENSP00000359612 | |
| Gorilla gorilla gorilla | ENSGGOP00000011801 | |
| Pan troglodytes | ENSPTRP00000001578 | |
| Callithrix jacchus | ENSCJAP00000046194 | |
| Pongo abelii | | XP_002810704.1 |
| Nomascus leucogenys | ENSNLEP00000012221 | |
| Macaca mulatta | ENSMMUP00000007544 | |
| Dipodomys ordii | ENSDORP00000014040 | |
| Tupaia chinensis | | XP_006149503.1 |
| Vicugna pacos | | XP_006205994.1 |
| Sorex araneus | ENSSARP00000004540 | |
| Rattus norvegicus | | NP_112618.1 |
| Mus musculus | ENSMUSP00000029842 | |
| Myotis lucifugus | ENSMLUP00000015461 | |
| Oryctolagus cuniculus | ENSOCUP00000013920 | |
| Ochotona princeps | ENSOPRP00000010338 | |
| Bos taurus | ENSBTAP00000018398 | |
| Ovis aries | ENSOARP00000015341 | |
| Pteropus vampyrus | ENSPVAP00000002651 | |
| Ailuropoda melanoleuca | ENSAMEP00000018226 | |
| Canis lupus familiaris | ENSCAFP00000030083 | |
| Felis catus | ENSFCAP00000022552 | |
| Equus caballus | ENSECAP00000022572 | |
| Otolemur garnettii | ENSOGAP00000008911 | |
| Sus scrofa | | NP_001096683.1 |
| Tursiops truncatus | ENSTTRP00000001248 | |
| Loxodonta africana | ENSLAFP00000003045 | |
| Echinops telfairi | ENSETEP00000004326 | |
| Dasypus novemcinctus | | XP_004480954.1 |
| Cavia porcellus | ENSCPOP00000010610 | |
| Mustela putorius furo | ENSMPUP00000005407 | |
| Monodelphis domestica | ENSMODP00000000353 | |
| Sarcophilus harrisii | | XP_003767388.1 |
| Ornithorhynchus anatinus | | XP_001515760.1 |
| Taeniopygia guttata | | XP_002186994.1 |
| Ficedula albicollis | ENSFALP00000006480 | |
| Meleagris gallopavo | | XP_003208786.1 |
| Gallus gallus | ENSGALP00000041735 | |
| Pelodiscus sinensis | | XP_006120365.1 |
| Anolis carolinensis | ENSACAP00000012895 | |
| Xenopus tropicalis | ENSXETP00000006723 | |
| Latimeria chalumnae | ENSLACP00000019410 | |
| Lepisosteus oculatus | | XP_006634777.1 |
| Astyanax mexicanus | ENSAMXP00000001313 | |
| Danio rerio | ENSDARP00000103460 | |
| Gasterosteus aculeatus | ENSGACP00000022771 | |
| Oreochromis niloticus | ENSONIP00000011727 | |
| Takifugu rubripes | ENSTRUP00000027403 | |
